# Supplementary material for: Unraveling the Genetic Structure of the Coconut Scale Insect Pest (Aspidiotus rigidus Reyne) Outbreak Populations in the Philippines
Source: Insects. 2019 Oct 26;10(11):374. doi: 10.3390/insects10110374 (PMC6920999; doi:10.3390/insects10110374)
Supplement: Supplementary file 1 [file insects-10-00374-s001.pdf]

Research Title

Unraveling the genetic structure of the coconut scale insect pest (*Aspidiotus rigidus* Reyne) outbreak populations in the Philippines

Authors and Affiliations

Joeselle M. Serrana[1,3], Naoto Ishitani[1,3], Thaddeus M. Carvajal[1,3], Billy Joel M. Almarinez[2,3], Alberto T. Barrion[2,3], Divina M. Amalin[2,3] and Kozo Watanabe[1,3]

[1] Department of Civil and Environmental Engineering, Ehime University, Bunkyo-cho 3, Matsuyama, 790-8577, Japan
[2] Biology Department, College of Science, De La Salle University, 2401 Taft Avenue, Manila 1004, Philippines
[3] Biological Control Research Unit, Center for Natural Sciences and Environmental Research, De La Salle University, 2401 Taft Avenue, Manila 1004, Philippines

# Corresponding Author

Prof. Kozo Watanabe, PhD

Department of Civil and Environmental Engineering, Ehime University, Bunkyo-cho 3, Matsuyama, 790-8577, Japan
E-mail Address: watanabe_kozo@cee.ehime-u.ac.jp
Phone & Fax Number: +81 (0) 89 927 9847

ORCID ID

J. M. Serrana (0000-0002-6967-5407); T.M. Carvajal (0000-0001-5166-0058); B.J.M Almarinez (0000-0003-2562-9887); K. Watanabe (0000-0002-7062-595X)

Supplementary Figure

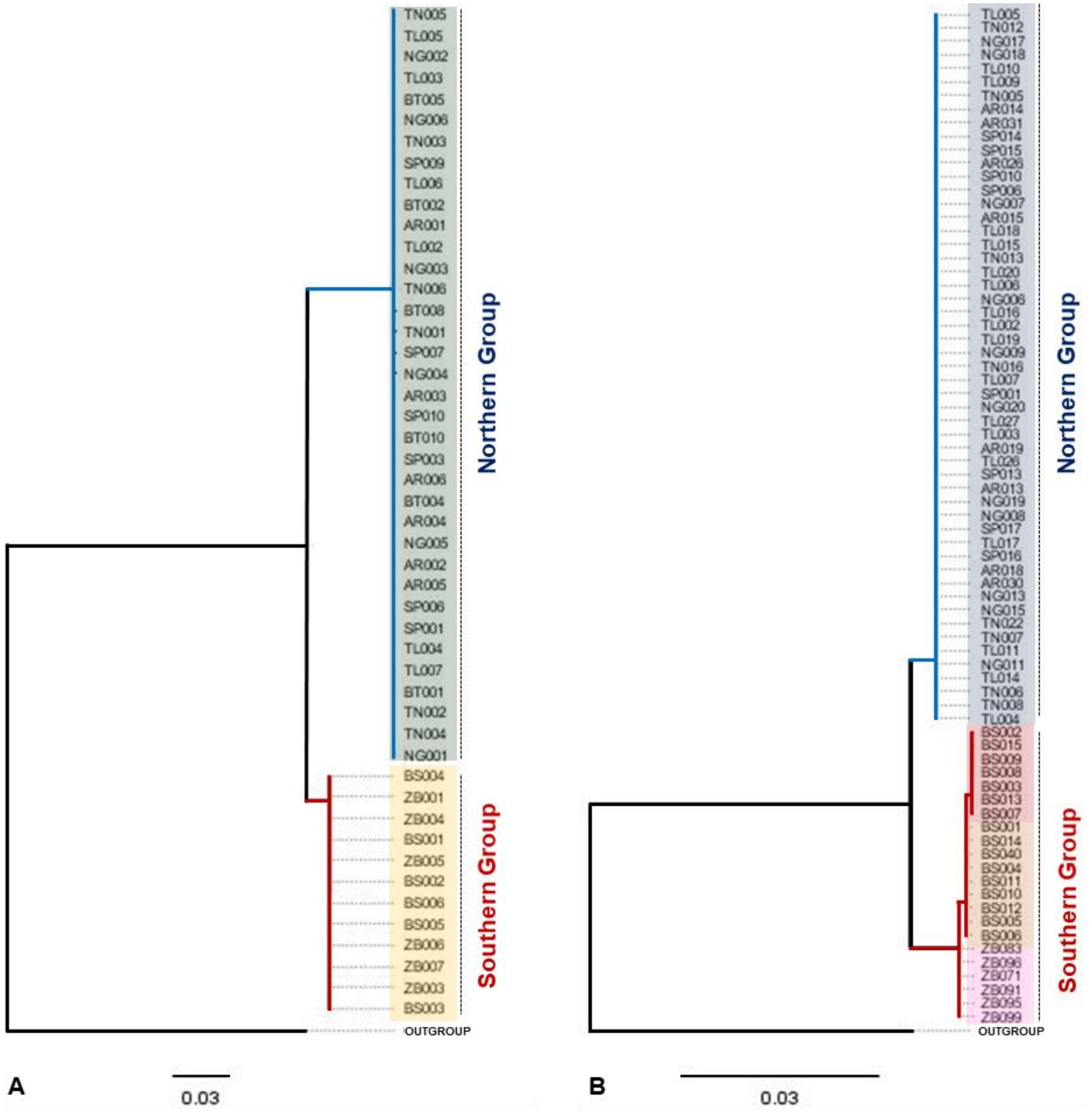

Fig. S1. Phylogeny of the outbreak *Aspidiotus rigidus* Reyne populations in the Philippines based on *mtCOI* (A) and *EF-1*α (B) sequences inferred using maximum likelihood. Refer to Table 1 for the corresponding code of each locality (e.g., BS003 sample collected from Basilan). Scale bar represents substitutions per site. Colors represent different haplotypes: *mtCOI* = 2; *EF-1*α = 4. Sequence of the morphologically similar coconut scale insect species, *Aspidiotus destructor* Signor*et al*so collected in the Philippines were included as outgroups.

Supplementary Tables

Table S1. Summary of the computations of the parameters for demographic expansion for the EF-1α dataset. The variance of the mismatch distribution is too small for the mtCOI dataset so no demographic parameters were estimated.

Statistics	AR	NG	SP	TL	TN	BS	ZB	Mean	s.d.
Tau	0.0000	0.0000	0.0000	0.0000	0.0000	0.7500	0.0000	0.1071	0.2835
Tau qt 2.50%	0.0000	0.0000	0.0000	0.0000	0.0000	0.0000	0.0000	0.0000	0.0000
Tau qt 5%	0.0000	0.0000	0.0000	0.0000	0.0000	0.0000	0.0000	0.0000	0.0000
Tau qt 95%	0.0000	0.0000	0.0000	0.0000	0.0000	0.0000	0.0000	0.0000	0.0000
Tau qt 97.50%	0.0000	0.0000	0.0000	0.0000	0.0000	0.0000	0.0000	0.0000	0.0000
Thetaθ	0.0000	0.0000	0.0000	0.0000	0.0000	0.0563	0.0000	0.0080	0.0213
Thetaθ qt 2.50%	0.0000	0.0000	0.0000	0.0000	0.0000	0.0000	0.0000	0.0000	0.0000
Thetaθ qt 5%	0.0000	0.0000	0.0000	0.0000	0.0000	0.0000	0.0000	0.0000	0.0000
Thetaθ qt 95%	0.0000	0.0000	0.0000	0.0000	0.0000	0.0000	0.0000	0.0000	0.0000
Thetaθ qt 97.50%	0.0000	0.0000	0.0000	0.0000	0.0000	0.0000	0.0000	0.0000	0.0000
Theta1	0.0000	0.0000	0.0000	0.0000	0.0000	6833.4477	0.0000	976.2068	2582.8005
Theta1 qt 2.50%	0.0000	0.0000	0.0000	0.0000	0.0000	6823.4477	0.0000	974.7782	2579.0208
Theta1 qt 5%	0.0000	0.0000	0.0000	0.0000	0.0000	6823.4477	0.0000	974.7782	2579.0208
Theta1 qt 95%	0.0000	0.0000	0.0000	0.0000	0.0000	6823.4477	0.0000	974.7782	2579.0208
Theta1 qt 97.50%	0.0000	0.0000	0.0000	0.0000	0.0000	6823.4477	0.0000	974.7782	2579.0208
SSD	0.0000	0.0000	0.0000	0.0000	0.0000	0.0283	0.0000	0.0040	0.0107
Model (SSD) p-value	0.0000	0.0000	0.0000	0.0000	0.0000	0.0000	0.0000	0.0000	0.0000
Raggedness index	0.0000	0.0000	0.0000	0.0000	0.0000	0.2781	0.0000	0.0397	0.1051
Raggedness p-value	0.0000	0.0000	0.0000	0.0000	0.0000	1.0000	0.0000	0.1429	0.3780

Table S2. Population pairwise F_{ST} values between the outbreak *Aspidiotus rigidus* Reyne populations for mtCOI and EF-1α data. Bold values are statistically significant at 0.05. Population code: AR, reared *A. rigidus* samples; BT, Bataan; NG, Nagcarlan; SP, San Pablo; TL, Talisay; TN, Tanauan; BS, Basilan; ZB, Zamboanga.

mtCOI	AR	BT	NG	SP	TL	TN	BS	ZB
**AR**								
**BT**	0.0000							
**NG**	0.0000	0.0000						
**SP**	0.0000	0.0000	0.0000					
**TL**	0.0000	0.0000	0.0000	0.0000				
**TN**	0.0000	0.0000	0.0000	0.0000	0.0000			
**BS**	**1.0000**	**1.0000**	**1.0000**	**1.0000**	**1.0000**	**1.0000**		
**ZB**	**1.0000**	**1.0000**	**1.0000**	**1.0000**	**1.0000**	**1.0000**	0.0000	

EF-1a	AR	NG	SP	TL	TN	BS	ZB
**AR**							
**NG**	0.0000						
**SP**	0.0000	0.0000					
**TL**	0.0000	0.0000	0.0000				
**TN**	0.0000	0.0000	0.0000	0.0000			
**BS**	**0.9738**	**0.9769**	**0.9738**	**0.9818**	**0.9738**		
**ZB**	**1.0000**	**1.0000**	**1.0000**	**1.0000**	**1.0000**	**0.7443**	

Table S3. Bayes factors and log marginal likelihoods. The mod.rank indicates model ranking with “1” as the best model; mod.prob the value of probability.

model		Specification	lmL	LBF	mod.rank	mod.prob
mtCOI	1	xxxx	-1522.21	-912.78	7	0.0000
	2	x0xx	-1457.69	-783.74	5	0.0000
	3	xx0x	-1452.44	-773.23	4	0.0000
	4	x	-1809.00	-1486.35	8	0.0000
	5	x0Dx	-1472.43	-813.22	6	0.0000
	6	xD0x	-1433.60	-735.55	3	0.0000
	**7**	**x0dx**	**-1065.82**	**0.00**	**1**	**0.9960**
	8	xd0x	-1068.64	-5.63	2	0.0040
EF-1a	1	xxxx	-1595.40	-38.70	5	0.0000
	2	x0xx	-1586.16	-20.22	2	0.0000
	**3**	**xx0x**	**-1576.05**	**0.00**	**1**	**1.0000**
	4	x	-1596.00	-39.90	6	0.0000
	5	x0Dx	-1587.30	-22.50	3	0.0000
	6	xD0x	-1591.08	-30.07	4	0.0000
	7	x0dx	-1643.81	-135.51	7	0.0000
	8	xd0x	-1654.40	-156.70	8	0.0000
